# Lipi Guben decoction in treating diarrheal irritable bowel syndrome: A study protocol for a randomized controlled trial

**DOI:** 10.1097/MD.0000000000023887

**Published:** 2021-01-22

**Authors:** Hongmei Yang, Xin Liu, Wei Peng, Rong Chen, Yang Chen

**Affiliations:** a The First Affiliated Hospital of Guizhou University of Traditional Chinese Medicine; b The Chinese People's Liberation Army 921 Hospital of the Joint Logistics Support Force; c Guiyang Nursing Vocational College.

**Keywords:** Lipi Guben Decoction, irritable bowel syndrome, research protocol, clinical efficacy

## Abstract

**Background:**

Irritable bowel syndrome (IBS) is a common functional bowel disorder. The global incidence of IBS is as high as 9% to 23%, accounting for about 50% of outpatients in gastroenterology, and the new case detection rate is 0.2% every year. IBS has become a global gastrointestinal functional disease. Although IBS is not a life-threatening disease, it seriously affects the quality of life of patients, causing huge economic and mental burden to individuals, society and families. Lipi Guben decoction (LPGBD) is an important auxiliary treatment for IBS, but lack of robust Evidence-based medicine evidence proving its efficacy. Therefore, we designed a randomized controlled trial to evaluate the efficacy and safety of LPGBD in the treatment of IBS.

**Methods:**

In this randomized controlled trial, a total of 100 eligible patients will be allocated to the blank control group or LPGBD group in a ratio of 1:1. The treatment period was 12 weeks. The primary outcome measure will be the total clinical effective rate. The Secondary outcomes will include IBS clinical symptom scores, IBS-Severity Scoring System, IBS-Quality of life, Hamilton Rating Scale for Anxiety, Hamilton Rating Scale for Depression, and Bristol Stool Form Scale. The safety outcome will include Echocardiogram, blood examination (including blood routine test, liver function test, and renal function test), urine routine test and stool routine test. The evaluation indicators and all safety results will be performed at baseline, week 4, week 8 and week 12.

**Results:**

This study will be helpful to evaluate the efficacy and safety of LPGBD in the treatment of IBS.

**Conclusion:**

LPGBD may improve the clinical efficacy of patients with IBS, which has important value in practical application

**Trial registration:**

Chictr20000039617, registration time: November 3, 2020

## 1 Introduction

Irritable bowel syndrome (IBS) is a common intermittent or durative gastrointestinal functional disease characterized by abdominal pain, abdominal distension with defecation habit change, abnormal stool characteristics without organic causes.^[1]^ The global incidence of IBS is as high as 9% to 23%, accounting for about 50% of outpatients in gastroenterology, and the new case detection rate is 0.2% every year. IBS has become a growing global public health issue.^[2]^ Although IBS is not a life-threatening disease, it seriously affects the quality of life of patients, and creates a huge economic and mental burden for affected individuals, society and families. IBS have been ranked as a common cause of illness-related absenteeism, second only to the common cold. According to the study, the direct and indirect costs of each IBS patient in the United States reach US $4527 per year.^[3]^

The symptoms of IBS are complex and diverse, and the condition is complicated with an unknown etiology and pathogenesis. It is generally believed that the occurrence of IBS is the result of a combination of multiple factors, including mental and psychological disorders, visceral hypersensitivity, gastrointestinal motility disorder and intestinal infection.^[4–6]^ Clinical studies have found that diarrhoea-predominant IBS (IBS-D) is the most common type.^[6–8]^ In modern medicine, drugs that regulates intestinal movement, reduces visceral hypersensitivity, improve central emotion, and regulate intestinal flora are commonly used to relieve IBS-D symptoms.^[9–12]^ Alosetron, a selective 5-hydroxytryptamine (5-HT) receptor blocker, was once considered to be the most effective drug for IBS-D, which was approved by Food and Drug Administration (FDA). However, due to its serious side effects, it was limited to female IBS patients with severe symptoms. Although the newly developed new drugs for IBS, such as zeamak and Trevor, have certain curative effect, their clinical application is limited due to their high side effects and high price. At present, modern medicine lacks specific and effective curative drugs for the treatment of IBS. The efficacy and mechanism of traditional Chinese medicine in the treatment of IBS have attracted more and more attention.^[13–15]^

Integrated traditional Chinese medicine (TCM) and Western medicine therapy has been the most distinctive methods in the treatment of IBS in China.^[16,17]^ Integrated TCM and Western medicine therapy for IBS patients could reduce symptoms, anxiety, depression, and improve quality of life.^[18–23]^ However, due to the small sample size and unscientific research methods, the conclusions are limited. Therefore, we conducted a randomized controlled trial (RCT) to evaluate the efficacy of safety of Lipi Guben decoction (LPGBD) in the treatment of IBS.

## 2 Methods

### 2.1 Trial design and registration

**Figure 1:**
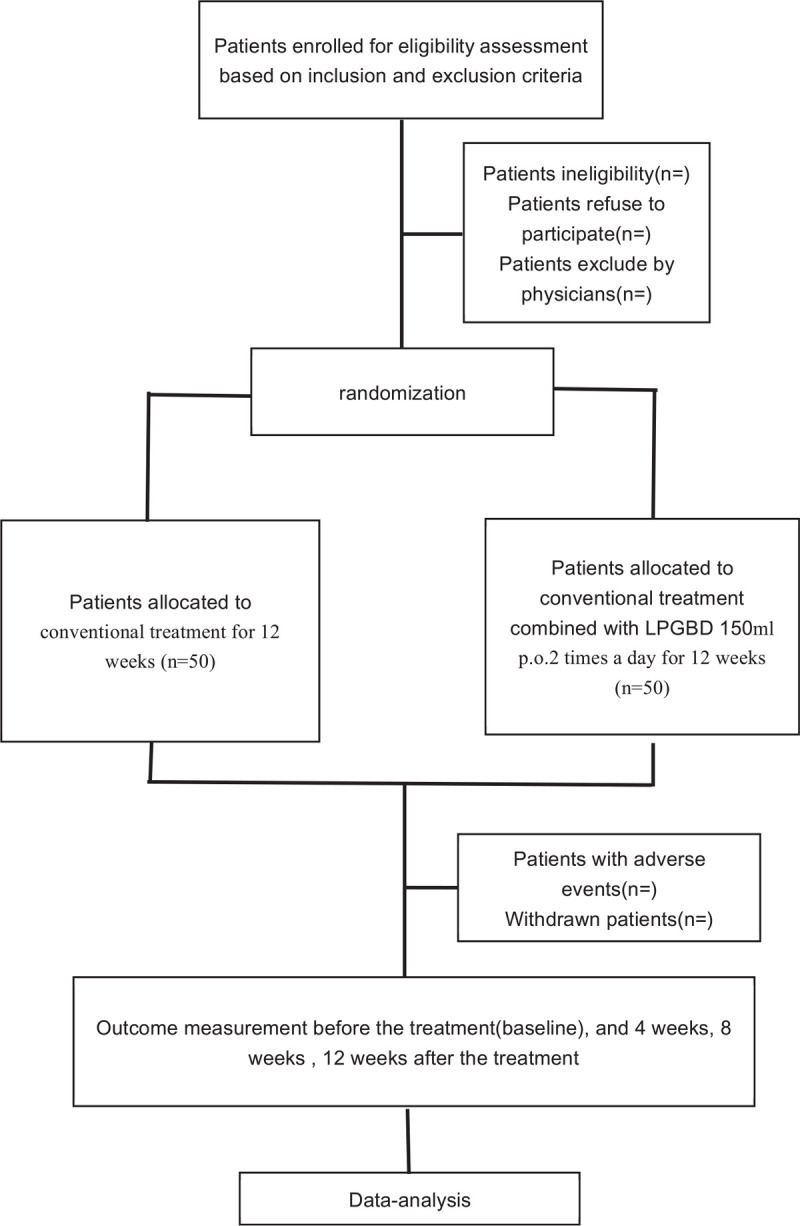
Study flow diagram.

**Figure 2:**
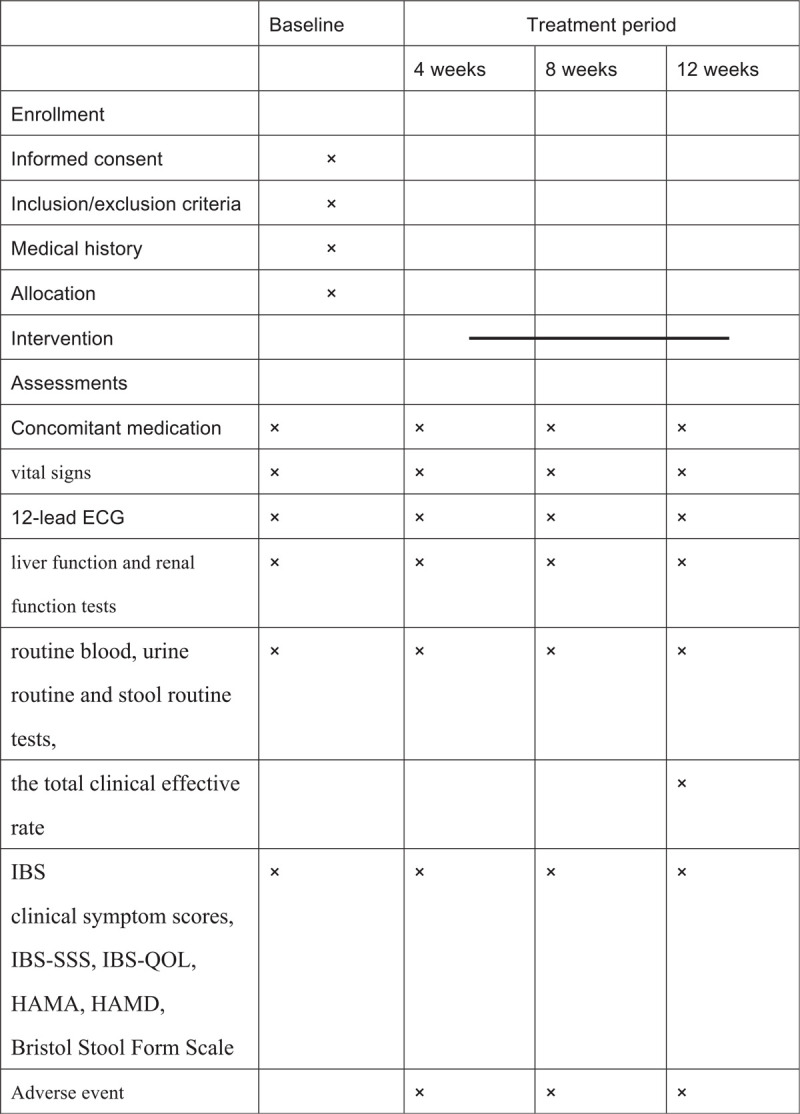
Study design schedule.

This is a RCT. Participants will be recruited from the outpatient clinics of the First Affiliated Hospital of Guizhou University of TCM. The Figure 1 shows the study design in the flowchart, and the Figure 2 illustrates the time schedule of enrolment, interventions, assessments, and visits of participants. The reporting of this trial is conducted according to the Standard Protocol Items: Recommendations for Intervention Trials (SPIRIT) guidelines (Additional file 1).

This protocol has been registered in the Chinese Clinical Trial Registry on November 3,2020, http://www.chictr.org.cn/edit.aspx?pid=63618&htm=4. And the registration number is ChiCTR2000039617.

### 2.2 Patient population and eligibility criteria

**Inclusion criteria:**

(1) Patients meet the the Rome III diagnostic criteria for IBS-D;
(2) Those who agree to participate in this study and are willing to sign informed consent.
(3) The age ranged from 18 to 65 years old.

**Exclusion criteria:**

(1) Under 18 years old or over 65 years old;
(2) A woman who is pregnant or is preparing for pregnancy, and lactating.
(3) Intestinal organic diseases, such as inflammatory bowel disease, intestinal tuberculosis, colon polyps;
(4) Patients with severe cardiovascular and cerebrovascular diseases, liver, kidney, hematopoietic system diseases and tumors;
(5) There were records of digestive tract operation;
(6) Patients with severe primary diseases and mental disorders;
(7) Patients with allergic history or drug and food allergy during the test period;
(8) Those who do not sign informed consent
(9) Those who do not meet the inclusion criteria, fail to use drugs according to the regulations, cannot judge the curative effect or incomplete data, which affects the evaluation of efficacy or safety.
(10) The researchers consider it inappropriate to conduct this clinical trial.

### 2.3 Sample size

The sample size was calculated based on the total clinical effective rate at 12 weeks. According to the previous literature,^[24]^ we hypothesized that the total clinical effective rate of the blank control group and LPGBD group at 12 weeks will be 73% and 93%, respectively. Set a=0.25 (2-sided) and b = 0.10. We use PASS 15 software (PASS 15.0.5 NCSs, LLC, USA) to calculate the size of the blank control group and LPGBD group N1 = N2 = 41. Assuming that 20% of patients may be lost during follow-up, a total of 100 patients will be recruited.

### 2.4 Randomization

Patients who met the inclusion criteria will be randomly assigned to blank control group and LPGBD group in the ratio of 1:1. Measures to Reduce the Risk of Bias Concealed randomization prevents selection bias. The random allocation sequence listing was generated by a statistician who will not participate in the clinical study using SAS v9.4 (SAS Institute, Cary, NC).5-10 minutes before treatment, the doctor will inform the patient about the randomization procedure, telephone number and name; then, the doctor will be informed of the random number of patients and the treatment group. The randomization information will be concealed until the end of the trial.

### 2.5 Blinding

Due to the nature of TCM, doctors and patients will know the grouping information. However, principal investigator, statisticians, and outcome evaluators will be blinded to group allocation.

### 2.6 Researchers

Five principal investigator with TCM background will be responsible for data collection. The supervisors and analysts who do not know the regulatory data will monitor the entire experiment. The principal investigator, outcome evaluators, and statisticians will be strictly trained before trial.

### 2.7 Recruitment

Patients will be recruited in the gastroenterology outpatient clinics of the First Affiliated Hospital of Guizhou University of TCM. The poster will briefly describe the trial and provide treatment details, as well as contact information. If a potential participant is interested in taking part, they can directly contact the study team. We will provide potential participants with detailed information about benefits and possible risks of the trial. If they decide to participate, they will be asked to sign written informed consent. If a participant withdraws from the trial, the reason for the withdrawal will be recorded. In order to improve the compliance of patients, we will strengthen doctor-patient communication and timely health education for patients.

### 2.8 Patient safety

Potential adverse events associated TCM include rash, skin allergy and liver and kidney function damage. Clinicians record the occurrence of any adverse events and the intervention will be stopped immediately. The independent safety supervision committee composed of 3 experts from different fields in the First Affiliated Hospital of Guizhou University of TCM has the right to terminate the study.

## 3 Interventions

### 3.1 The blank control group

Subjects in the blank control group will receive conventional treatment: Pinaverium Bromide Tablets (50 mg), orally 3 times daily for 12weeks; Bifidobacterium triple viable capsule (420 mg), orally twice times daily for 12weeks.

### 3.2 The LPGBD group

Subjects in the LPGBD group will be given LPGBD, based on the conventional treatment in the blank control group. The herbal formula of LPGBD comprises the following TCM: fried Atractylodes macrocephala 15 g, Poria cocos 10 g, tangerine peel 6G, Rhizoma Pinelliae 10 g, Shenqu 10 g, malt 15 g, roasted licorice 6G, Astragalus 20 g, Paeonia Alba 10 g. The medicinal materials were decocted twice at 100 °C under a normal pressure, each for 40 minutes. Each decoction (150 ml) should be taken twice a day.

### 3.3 The course of treatment

The duration of treatment was 12 weeks.

### 3.4 Combined treatment regulations

During the trial, Participants are prohibited from taking any other TCM, western medicine or therapeutic measures (such as acupuncture, cupping, and massage). If a concomitant intervention is used, the name, dosage, and frequency of the accompanying interventions should be recorded in detail.

## 4 Outcome measures

### 4.1 Primary outcome

Disease outcomes include cure, significant effect, effective, and ineffective. The main outcome will be the total clinical effective rate of the 2 groups after treatment (week 12), that is, that is, the sum of cure rate, apparent efficiency and effective. Outcome indicators will be collected at the 12th week.

### 4.2 Secondary outcomes

(1) IBS clinical symptom scores: abdominal pain, diarrhea, abdominal distension will be scored as follows: 0, none;2, light;4, moderate; 6, heavy. IBS clinical symptom scores will be measured on week 0, 4,8, 12.
(2) IBS-Quality of life (IBS-QOL): IBS-QOL consists of 8 domains: dysphoria, interference with activity, body image, health worry, food avoidance, social reaction, sexual function and impact on relations. The IBS-QOL score ranged from 0 to 100 points and a higher score indicated a better QOL. IBS-QOL will be measured on week 0, 4,8, 12.
(3) IBS-Severity Scoring System (IBS-SSS): The IBS-SSS score ranged from 0 to 500 points and a higher score indicated a worse SSS. The IBS-SSS are scored as follows: mild, ≤175 points; moderate severity, >175 points and ≤300 points; severe, >300 points. IBS-SSS will be measured on week 0, 4,8, 12.
(4) Hamilton Rating Scale for Anxiety (HAMA): HAMA are scored as follows: normal, <7 points; suspected anxiety, 7 to 14 points; definite anxiety, 14 to 21 points; obvious anxiety, 21 to 29 points; serious anxiety, ≥29 points. HAMA will be measured on week 0, 4,8, 12.
(5) Hamilton Depression Scale: HAMA are scored as follows: normal, <8 points; suspected depression, 8-20 points; definite depression, 20 to 35 points; severe depression, >35 points. Hamilton Rating Scale for Depression will be measured on week 0, 4,8, 12.
(6) Bristol Stool Form Scale: Bristol Stool Form Scale will be measured on week 0, 4,8, 12.

### 4.3 Safety outcomes

Safety results will include echocardiogram, blood tests (including routine blood tests, liver function tests and kidneys function tests), urine routine tests and stool routine tests. All outcomes be measured on week 0, 4,8, 12. If an adverse event occurs, the investigator will record the details (including symptoms, time of occurrence, duration, examination, and results) in the form of a case record form (CRF). Serious adverse reactions will be reported to the ethics committee of the First Affiliated Hospital of Guizhou University of TCM, and relevant rescue procedures will be started immediately.

### 4.4 Data management

Test data need to be recorded in paper-based and electronic CRF. An independent data-monitoring committee reviewed the data every 3 months. During the trial, only data managers and principal investigators will have access to the electronic CRF. At the end of the trial, the database will be locked by the data management team, after which the principal investigators cannot modify the data. Paper and electronic documents will be kept for at least 5 years. If readers and reviewers have any questions, they can contact the appropriate author to obtain the original content data.

## 5 Ethics

This study has been approved by the ethics committee of the First Affiliated Hospital of Guizhou University of TCM (ethical approval number: YE2020-126-11) and registered in the China clinical trial registration center on November 3, 2020. The trial strictly followed the Helsinki Declaration (2000 EDITION). Only patients with informed consent can participate in the trial.

## 6 Dissemination

The results of the study will be communicated to healthcare professionals, participants and the public through peer-reviewed publications, scientific conferences. Moreover, the data of this study can be obtained from corresponding authors according to reasonable requirements.

## 7 Confidentiality

All personal information of the participants will be kept strictly confidential and the anonymized individual patient data will be shared on request.

## 8 Ancillary and post-trial care

In case of direct injury related to this study, corresponding medical and nursing care can be obtained. Emergency contact number will be Provided for each subject so that they can contact the researcher whenever they have any questions or questions.

## 9 Statistical analysis

The clinical results will be analyzed by SPSS 22.0 statistical software (IBM SPSS statistics, IBM Corp, Somers, NY). In this study, the intention-to-treat analysis and per-protocol analysis will be conducted. Primary outcomes will be based on the per-protocol analysis, with the intention-to-treat analysis analysis conducted to assess robustness of the results. The measurement data will be expressed as mean ± standard deviation, and the counting data will be expressed as percentage, will be tested by a t test and chi-square test respectively. All statistical tests will be 2-sided and *P* values less than .05 will be considered to be statistically significant.

## 10 Discussion

The main clinical manifestations of IBS-D are abdominal pain and pathogenic factors, which seriously reduce the quality of life of patients and affect their work. The main therapeutic target of IBS-D is to reduce abdominal pain and diarrhea, improve gastrointestinal function and the quality of life of patients. There is no good treatment for IBS-D in modern medicine. Therefore, alternative therapy has been welcomed and focused on. TCM has been used in the treatment of IBS-D for hundreds of years a certain effect. However, in terms of evidence-based medicine, the quality of these studies was moderate. Therefore, in order to evaluate the efficacy and safety of LPGBD on IBS-D, we designed a RCT for 12 weeks to evaluate the efficacy and safety of LPGBD in the treatment of IBS-D.

RCT is the gold standard to evaluate the efficacy and safety of drugs. Of course, this study also has some shortcomings, mainly reflected in the following 2 aspects:

(1) Due to the long observation period of this study, it can be concluded that the shedding rate is high, which will affect the results of the study. Therefore, we should maintain a good relationship between doctors and patients in the research process, and the scale involved in this study is a self-evaluation scale, which can be conducted subjectively by patients, so as to better improve the quality of research data;
(2) The patients in this study are limited to Chinese patients, so the results of this study have some limitations and may not be extended to other regions.

## 11 Trial status

Protocol version number V1.0, September 10, 2020. The trial is currently in the recruitment phase of participants. Recruitment begins on November 10, 2020 and is expected to end on December 31, 2022.

## Acknowledgments

We would like to thank all the patients who will participate in the trial and the staff for their support.

## Author contributions

**Conceptualization:** Hongmei Yang, Xin Liu, Wei Peng, Rong Chen.

**Investigation:** Xin Liu.

**Supervision:** Yang Chen.

**Writing – original draft:** Hongmei Yang.

**Writing – review & editing:** Hongmei Yang, Yang Chen.
